# Sex-based differences in the association of leisure-time physical activity with the risk of depression: the Ansan and Ansung study of the Korean Genome and Epidemiology Study (KoGES)

**DOI:** 10.3389/fpubh.2023.1176879

**Published:** 2023-06-15

**Authors:** Jae Ho Park, Nam-Kyoo Lim, Hyun-Young Park

**Affiliations:** ^1^Division of Population Health Research, Department of Precision Medicine, Korea National Institute of Health, Korea Disease Control and Prevention Agency, Cheongju, Republic of Korea; ^2^Department of Precision Medicine, Korea National Institute of Health, Korea Disease Control and Prevention Agency, Cheongju, Republic of Korea

**Keywords:** depression, physical activity, resistance training, population study, prevention

## Abstract

**Objective:**

Depression is a serious mental disorder which is the leading cause of suicide. This study investigated the association between incident depression and 4-year leisure-time physical activity (PA) levels and/or resistance training (RT).

**Methods:**

This community-based Korean cohort included 3,967 participants without depression at baseline. The average PA-time (the total duration of moderate-intensity leisure-time PA) up to 4 years prior to baseline enrollment was calculated to evaluate the cumulative levels of PA. Participants were divided into four groups based on their average PA-time: “Non-PA,” “ <150 min/week,” “150–299 min/week,” and “≥300 min/week.” Furthermore, based on compliance to PA guidelines (≥150 min/week of PA-time) and participation in RT, the participants were categorized into four subgroups: “Low-PA,” “Low-PA+RT,” “High-PA,” and “High-PA+RT.” A multivariate Cox proportional hazards regression model was used to assess the 4-year incidence of depression according to leisure-time PA levels and/or regularity of RT.

**Results:**

During the mean 3.72 ± 0.69 years of follow-up, 432 participants (10.89%) developed depression. In women, performing 150–299 min/week of moderate-intensity leisure-time PA was associated with a 38% risk reduction for incident depression (HR, 0.62; CI, 0.43–0.89; *p* < 0.05), whereas more than 300 min/week of that was related to a 44% risk reduction for incident depression (HR, 0.56; CI, 0.35–0.89; *p* < 0.05) as compared to that in the Non-PA group. However, in men, there was no significant relationship between the amount of leisure-time PA per week and the risk of incident depression. Moreover, in both sexes, RT had no significant effect on depression in either the Low-PA or High-PA group.

**Conclusions:**

There was an inverse dose–response association between leisure-time PA levels and incident depression only in women, whereas adding RT to high levels of PA had no significant effect on depression in either sex.

## Introduction

Depression is a common but serious mental disorder. It is the leading cause of suicide; it significantly increases suicidal ideation and suicide attempts (1, 2). According to the World Health Organization (WHO), approximately 322 million people worldwide experienced depression in 2015, which increased by 18.4% from 2005 to 2015 (3). A recent systematic review reported that approximately 53.2 million people worldwide had depression due to the COVID-19 pandemic in 2020 (4). As a result, there is a growing need for strategies to prevent and manage mental disorders, including depression, and prevent life-threatening conditions.

The WHO and U.S. Department of Health and Human Services have recommended all adults to participate in moderate-intensity physical activity (PA) for at least 150 min per week to improve mental health by reducing symptoms of depression as well as anxiety (5, 6). In previous cross-sectional studies, adhering to the PA guideline was negatively associated with the risk of depression in both sexes (7). However, the association was significant only in women (8) or only in men (9), suggesting a sex-based difference in the association of leisure-time PA level with the risk of depression. Therefore, it is controversial whether the protective effect of increased leisure-time PA level on the risk of depression is the same in both sexes. Moreover, meta-analytical evidence has shown a significant relationship between satisfying the PA guideline and incident depression regardless of age and sex (10); however, the presence of an inverse dose–response association between leisure-time PA levels and the risk of depression remains controversial. This association was not significant in German (11) and Norwegian cohorts (12), but it was significant in a Mexican cohort (13). Potential differences among ethnicities and races remain to be investigated using other cohort data. Taken together, further evidence is required to determine the associations between leisure-time PA levels and incident depression.

Muscle-strengthening PA, academically known as resistance training (RT), is a type of leisure-time PA in which major muscle groups work against an external force or weight. Generally, regular RT ≥2 times per week is recommended to improve bone mass, muscle strength and mass, insulin sensitivity, and blood glucose levels (5, 14). In the United States, from 1998 to 2018, the prevalence of meeting RT recommendations increased from 17.7 to 27.6% (15). However, despite the increased interest in RT, its preventive effects against mental disorders have received less attention than those received by aerobic-related leisure-time PA. Although a recent cross-sectional study documented an inverse association between RT regularity and the risk of depression in a German cohort (16), further longitudinal studies are needed to examine the cause-and-effect associations between them. Evidence regarding sex-based differences in the relationship between RT participation and incident depression also remains lacking.

Therefore, the present study aimed to investigate the associations of depression with cumulative leisure-time PA level and RT regularity in Korean adults using a population-based prospective cohort. We further investigated for the presence of an inverse dose–response association between leisure-time PA levels and incident depression. Moreover, we examined whether RT had an additional protective effect against depression in participants with high leisure-time PA levels.

## Materials and methods

### Study participants

This study used data from the Ansan and Ansung cohort, which is part of the Korean Genome and Epidemiology Study (KoGES), an ongoing prospective population-based cohort study comprising participants from rural (Ansung) and urban (Ansan) areas in Gyeonggi-do, Korea. Detailed information on this cohort was provided in a previous study (17). We considered data of the seventh wave of the cohort as the baseline as incident depression was measured from the seventh wave. The 5,906 participants examined in 2013–2014 (seventh wave) were followed up biennially until 2017–2019 (ninth wave).

Among the 5,906 participants, those without data of the Korean version of the Geriatric Depression Scale-Short Form (SGDS-K) (*n* = 22), PA level (*n* = 172), and covariates (*n* = 192) and those who were diagnosed with dementia (*n* = 795) or depression (*n* = 758) at baseline were excluded. Overall, 3,967 participants (1,950 women) were included in the final analysis (Supplementary Figure 1). This study was approved by the Institutional Review Board Committee of the Korea National Institute of Health, Korea Disease Control and Prevention Agency (Approval No. 2021-04-02-P-A).

### Measurement of PA

**Figure 1:**
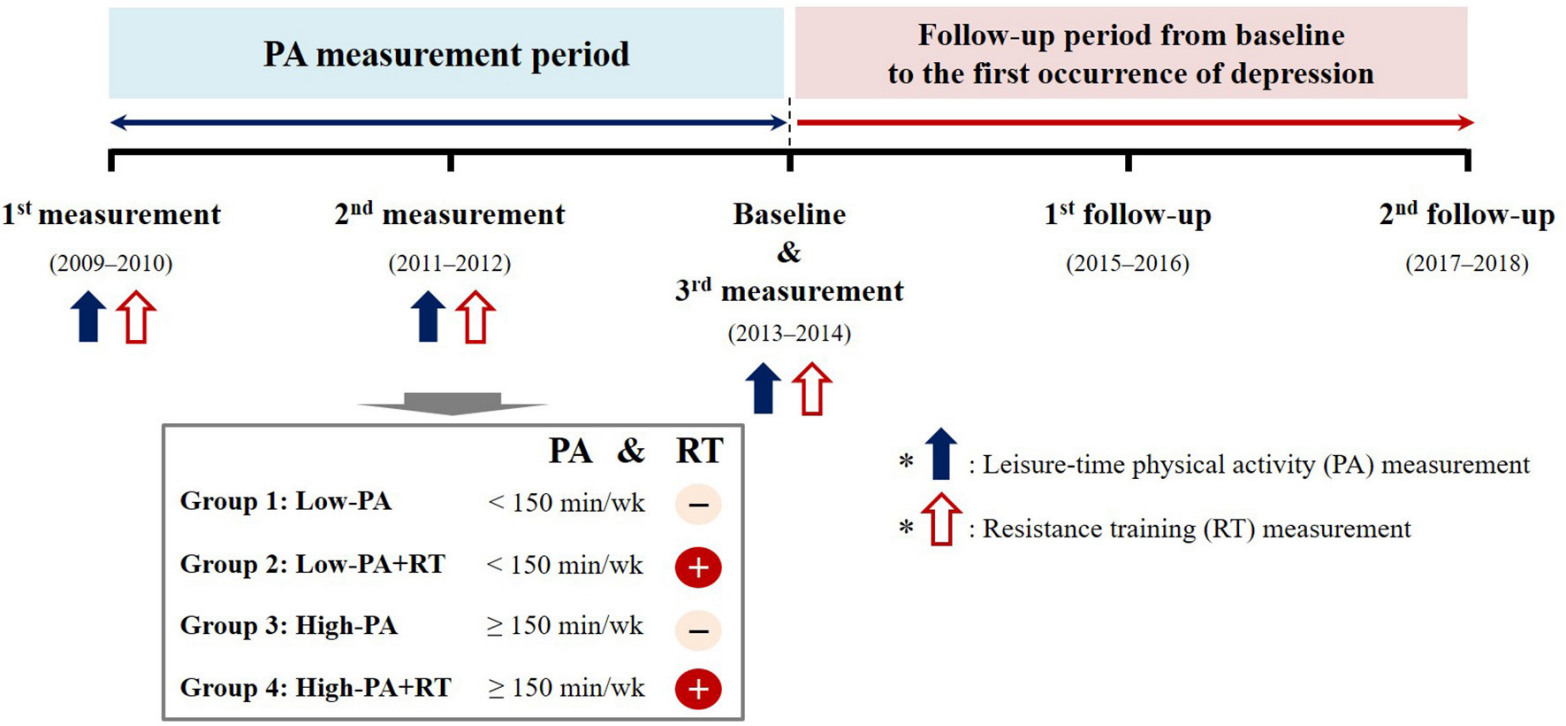
Scheme depicting the study grouping and follow-up over time.

Figure 1 illustrates the overall scheme of this study. All participants provided details of their regular leisure-time PA, including RT, during the 4-year PA measurement period. Intensity, frequency (per week), and duration (min) of leisure-time PA in a typical week were assessed. The average PA-time (the total duration of moderate-intensity leisure-time PA; min/week) during the 4 years prior to baseline enrollment was calculated to evaluate the cumulative level of PA over time. Moderate-intensity leisure-time PA was defined as participation in sports or exercise to the point of sweating. Further, the participants were classified into two groups according to the WHO recommendation, i.e., moderate-intensity leisure-time PA for at least 150 min per week, as: “Low-PA” (not following the recommendation) and “High-PA” (following the recommendation) (18). Furthermore, to investigate the presence of an inverse dose–response association between the levels of moderate-intensity leisure-time PA and incident depression, participants were divided into one of four subgroups based on the recent guideline (6): “Non-PA,” “ < 150 min/week,” “150–299 min/week,” and “≥300 min/week.”

RT was defined as any training program involving muscle contraction against external resistance using body weight, weight machines, barbells, and dumbbells. The frequency (per week) and training period (months) of the most recent evaluation during the PA measurement period were assessed to examine RT regularity. Regular RT was defined as participation in an RT program for more than 1 day per week. Participants were divided into four subgroups according to leisure-time PA levels and RT regularity: “Low-PA,” “Low-PA+RT,” “High-PA,” and “High-PA+RT.”

### Definition of incident depression

Incident depression was defined as the first occurrence of a score of ≥6 on the 15-item SGDS-K (*n* = 428) at any follow-up period or by diagnosis by a physician (*n* = 11). The SGDS-K is a valid and reliable instrument for screening depression in older individuals in Korea (19, 20). We used a score of 6 as the optimal cutoff point for screening both minor and major depressive disorders (21). The participants were divided into two groups based on new-onset depression during the follow-up period, “Non-DEP” (those without depression) and “DEP” (those with depression), to compare the baseline characteristics of the participants.

### Covariates

Sociodemographic and health-related factors, including age, sex, marital status, educational level, household income, drinking and smoking habits, PA-time, body mass index (BMI), waist circumference (WC), blood pressure (BP), hypertension, diabetes mellitus, and laboratory parameters, were included in our analyses. All covariates were derived from the baseline data, except for PA-time. Marital status was classified as “divorced/widowed/single” and “married/partnered.” Educational level was classified as elementary school graduate or lower, middle or high school graduate, and college graduate or higher. Household income was categorized as “ < 1,” “1 to < 2,” “2 to < 3,” “3 to < 4,” and “≥4” million KRW/month (million Korean won per month). Drinking and smoking habits were classified as “never,” “former,” and “current.” PA-time was defined as the average total time (min/week) of moderate-intensity leisure-time PA during the 4-year PA measurement period.

Anthropometric data, including body weight, height, and WC, were measured by trained healthcare providers using standardized protocols. BMI was calculated as body weight (kg) divided by height (m) squared (kg/m^2^). Trained healthcare providers also measured the BP using standard methods. Systolic BP (SBP) and diastolic BP (DBP) were defined as the average of two readings for the arm with the highest SBP that was obtained after resting for 5 min while seated. Blood samples were collected after fasting overnight for at least 8 h. Furthermore, biochemical assays were performed to determine total cholesterol (T-Chol), high-density lipoprotein cholesterol (HDL-C), triglyceride (TG), and fasting blood glucose (FBG) levels. The presence of hypertension was defined based on a previous diagnosis by a physician, the current use of antihypertensive drugs, SBP ≥140 mmHg, or DBP ≥90 mmHg. The presence of diabetes mellitus was defined based on a previous diagnosis by a physician; the current use of antidiabetic medications, including insulin and oral hypoglycemic agents; FBG ≥126 mg/dl; or, glycated hemoglobin ≥6.5%. A detailed description of the biochemical analyses is available elsewhere (17).

### Statistical analysis

All statistical analyses were conducted using SAS 9.4 (SAS Institute, Cary, North Carolina, United States). The baseline characteristics of the participants are presented as descriptive statistics. Continuous variables are presented as mean ± standard deviation, whereas categorical variables are expressed as absolute frequencies and percentages (%). The chi-square test was used to compare marital status, educational level, household income, drinking and smoking habits, participation in RT, and the prevalence of noncommunicable diseases (e.g., hypertension and diabetes mellitus) between the groups. Independent *t*-tests were used to compare age, PA-time, BMI, WC, SBP, DBP, T-Chol, HDL-C, TG, and FBG levels between the groups.

A multivariate Cox proportional hazards regression model was used to evaluate the hazard ratios (HRs) and 95% confidence intervals (CIs) for the incidence of depression. The models were adjusted for age, sex, drinking and smoking habits, educational level, marital status, household income, BMI, hypertension, and diabetes mellitus. We performed a subgroup analysis on the association between leisure-time PA levels and incident depression by age (< 65 and ≥65 years), educational level (≤ middle school and ≥high school), household income (< 3 and ≥3 million KRW/month), BMI (< 25 and ≥25 kg/m^2^), current drinking habit (no and yes), smoking status (never and ever), hypertension (no and yes), and diabetes mellitus (no and yes) for each sex. The *p*-value for the interaction was estimated to investigate the consistency of patterns in associations across subgroups for each sex. All tests were two-tailed, and statistical significance was set at a *p* < 0.05.

## Results

A total of 3,967 participants were enrolled. The mean follow-up period was 3.72 ± 0.69 years (range 1.50–4.67 years). During the follow-up period, 432 participants (10.89%) developed depression. Table 1 shows the baseline characteristics of the participants based on their leisure-time PA levels and sex. In both sexes, the mean age, WC, SBP, DBP, and prevalence of a low educational level (≤ elementary school) were markedly lower in the High-PA group than those in the Low-PA group. A high household income (≥4 million KRW/month) was significantly more common in the High-PA group than that in the Low-PA group in both men and women. Among men, the proportion of current smokers was higher and the prevalence of diabetes mellitus was lower in the Low-PA group than those in the High-PA group. In women, compared to the Low-PA group, the High-PA group was significantly associated with higher T-Chol and HDL-C levels and higher proportions of those living with a partner (married or partnered) and current drinkers; however, it had a lower prevalence of hypertension.

**Table 1:** Baseline characteristics of study participants based on leisure-time PA levels.

**Variables**	**Men (*****n*** = **2,017)**		***p*-value**	**Women (*****n*** = **1,950)**		***p*-value**
	**Low-PA (*****n*** = **998)**	**High-PA (*****n*** = **1,079)**		**Low-PA(*****n*** = **1,128)**	**High-PA (*****n*** = **822)**	
**Age** (years)	62.11 ± 7.91	60.85 ± 7.28	< 0.001	62.89 ± 8.26	60.16 ± 7.16	< 0.0001
**Marital status**, *n* (%)			0.92			< 0.05
Divorced/widowed/single	48 (4.81)	48 (4.71)		239 (21.19)	143 (17.40)	
Married/partnered	950 (95.19)	971 (95.29)		889 (78.81)	679 (82.60)	
**Educational level**, *n* (%)			< 0.0001			< 0.0001
≤ Elementary school	178 (17.83)	97 (9.52)		453 (40.16)	201 (24.45)	
Middle/high school	690 (69.14)	676 (66.34)		620 (54.96)	566 (68.86)	
≥ College	130 (13.03)	246 (24.14)		55 (4.88)	55 (6.69)	
**Household income**, *n* (%)			< 0.0001			< 0.0001
< 1 (million KRW/month)	233 (23.35)	120 (11.78)		404 (35.82)	174 (21.17)	
1 to < 2	199 (19.94)	146 (14.33)		229 (20.30)	147 (17.88)	
2 to < 3	176 (17.63)	161 (15.80)		192 (17.02)	130 (15.81)	
3 to < 4	145 (14.53)	175 (17.17)		123 (10.90)	137 (16.67)	
≥ 4	245 (24.55)	417 (40.92)		180 (15.96)	234 (28.47)	
**Drinking habit**, *n* (%)			0.31			< 0.01
Never drinker	203 (20.34)	209 (20.51)		883 (78.28)	592 (72.02)	
Ex-drinker	138 (13.83)	118 (11.58)		30 (2.66)	17 (2.07)	
Current drinker	657 (65.83)	692 (67.91)		215 (19.06)	213 (25.91)	
**Smoking habit**, *n* (%)			< 0.0001			0.50
Never smoker	225 (22.55)	260 (25.51)		1,116 (98.94)	811 (98.66)	
Ex-smoker	462 (46.29)	548 (53.78)		3 (0.26)	5 (0.61)	
Current smoker	311 (31.16)	211 (20.71)		9 (0.80)	6 (0.73)	
**PA-time** (min/week)	46.33 ± 54.99	330.12 ± 177.55	< 0.0001	57.70 ± 55.55	290.02 ± 134.58	< 0.0001
**RT**, *n* (%)	48 (4.81)	263 (25.81)	< 0.0001	30 (2.66)	140 (17.03)	< 0.0001
**BMI** (kg/m^2^)	24.33 ± 3.07	24.54 ± 2.68	0.09	24.79 ± 3.41	24.60 ± 3.08	0.20
**WC** (cm)	88.28 ± 8.60	86.72 ± 7.97	< 0.0001	85.76 ± 9.75	83.17 ± 9.06	< 0.0001
**SBP** (mmHg)	121.64 ± 14.72	119.49 ± 14.33	< 0.001	120.51 ± 17.01	116.57 ± 16.04	< 0.0001
**DBP** (mmHg)	79.52 ± 9.70	77.83 ± 9.53	< 0.0001	75.73 ± 9.54	74.43 ± 9.66	< 0.01
**T-Chol** (mg/dl)	183.06 ± 33.45	182.70 ± 33.49	0.81	191.90 ± 34.69	196.09 ± 35.69	< 0.01
**HDL-C** (mg/dl)	43.14 ± 11.00	43.73 ± 11.48	0.24	47.27 ± 11.13	49.31 ± 11.83	< 0.001
**TG** (mg/dl)	150.99 ± 111.02	145.12 ± 106.89	0.23	128.88 ± 73.19	123.97 ± 67.81	0.13
**FBG** (mg/dl)	99.38 ± 21.26	99.97 ± 21.45	0.53	95.01 ± 19.78	94.25 ± 19.08	0.39
**Hypertension**, *n* (%)	469 (46.99)	469 (46.03)	0.66	519 (46.01)	319 (38.81)	< 0.01
**Diabetes mellitus**, *n* (%)	161 (16.13)	207 (20.31)	< 0.05	189 (16.76)	116 (14.11)	0.11

PA, physical activity; KRW, Korean won; PA-time, total time of regular participation in any sports or exercise to the point of sweating; RT, resistance training; BMI, body mass index; WC, waist circumference; SBP, systolic blood pressure; DBP, diastolic blood pressure; T-Chol, total cholesterol; HDL-C, high-density lipoprotein cholesterol; TG, triglycerides; FBG, fasting blood glucose.

Baseline characteristics of the study participants based on sex and new-onset depression during the follow-up period are presented in Supplementary Table 1. The incidence rate of depression was significantly higher in women (12.67%) than that in men (9.17%) (*p* < 0.001). In both sexes, the mean age and the prevalence of a low educational level (≤ elementary school) were significantly higher in the DEP group than those in the Non-DEP group, whereas PA-time and the proportion of a high household income (≥4 million KRW/month) were markedly higher in the Non-DEP group than those in the DEP group. Among men, the proportion of current smokers was markedly higher in the DEP group than that in the Non-DEP group, whereas the proportion of those performing regular RT was significantly higher in the Non-DEP group than that in the DEP group. In women, compared to the Non-DEP group, the DEP group had significantly higher WC, SBP, DBP, and prevalence of hypertension but a lower T-Chol level.

**Figure 2:**
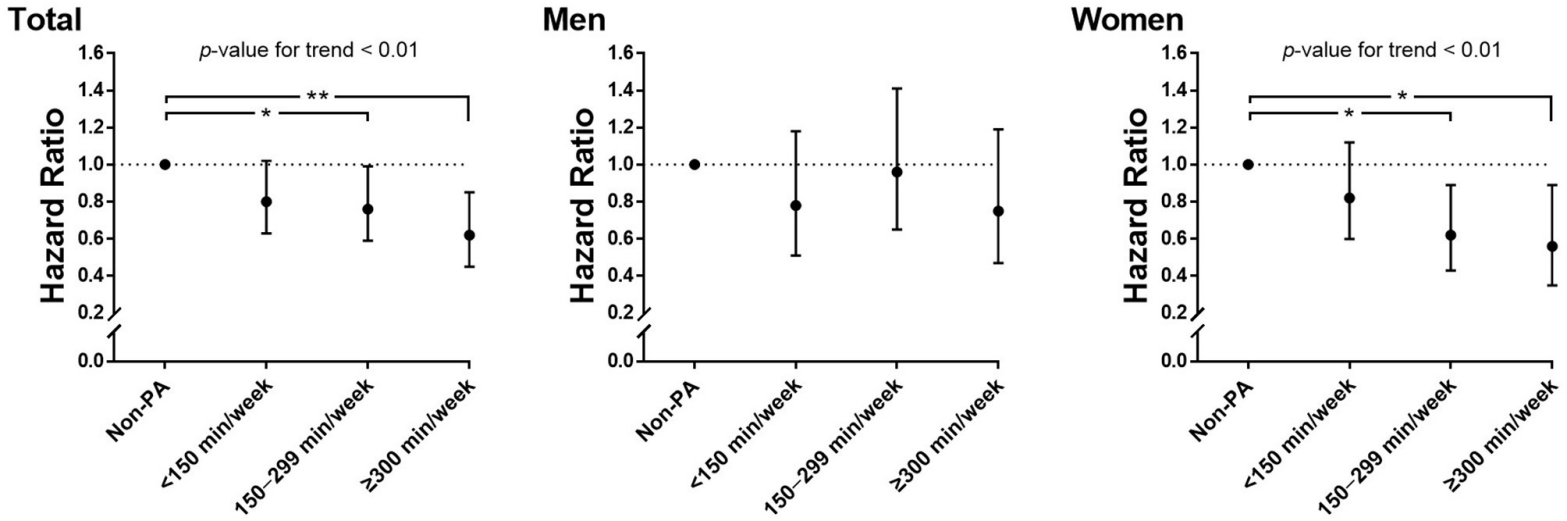
Hazard ratios for new-onset depression according to leisure-time PA per week and sex. Adjusted for age, sex, drinking, smoking, educational level, marital status, household income, BMI, hypertension, and diabetes mellitus. PA, physical activity; BMI, body mass index; ^*^, *p* < 0.05; ^**^, *p* < 0.01.

Table 2 presents the association between leisure-time PA levels and the risk of incident depression. A high level of leisure-time PA was related to a reduced depression risk in 33% of women (*p* < 0.01), but not in men. We further investigated the presence of an inverse dose–response association between the amount of leisure-time PA per week and the risk of incident depression (Figure 2). In women, performing moderate-intensity leisure-time PA for 150–299 min/week was associated with a 38% risk reduction for incident depression (HR, 0.62; CI, 0.43–0.89; p < 0.05), whereas performing more than 300 min/week was related to a 44% risk reduction for incident depression (HR, 0.56; CI, 0.35–0.89; *p* < 0.05). In women, although our analysis revealed no significant difference in the risk of depression between the 150–299 min/week and ≥300 min/week groups (*p* = 0.55), there was an inverse dose–response association between leisure-time PA level and the risk of depression after adjusting for covariates (*p* for trend < 0.01). However, in men, there was no significant relationship between the amount of leisure-time PA per week and the risk of incident depression.

**Table 2:** Hazard ratios for new-onset depression based on leisure-time PA levels by sex.

	***N***	**PA-time (min/week)**	**Total person-years**	**Participants with depression, *n* (%)**	**Event rate (1,000-person year)**	**Crude Model**	**Model 1**	**Model 2**
						**HR (95% CI)**	**HR (95% CI)**	**HR (95% CI)**
**Total**								
Low-PA	2,126	52.36 ± 55.56	7468.91	278 (13.08)	37.22	1 (reference)	1 (reference)	1 (reference)
High-PA	1,841	312.22 ± 160.99	6645.51	154 (8.37)	23.17	0.63 (0.52–0.77) ^****^	0.79 (0.64–0.97) ^*^	0.79 (0.64–0.97) ^*^
**Men**								
Low-PA	998	46.33 ± 54.99	3506.58	104 (10.42)	29.66	1 (reference)	1 (reference)	1 (reference)
High-PA	1,019	330.12 ± 177.55	3638.34	81 (7.95)	22.26	0.76 (0.57–1.02)	0.96 (0.71–1.30)	0.97 (0.71–1.32)
**Women**								
Low-PA	1,128	57.70 ± 55.55	3962.33	174 (15.43)	43.91	1 (reference)	1 (reference)	1 (reference)
High-PA	822	290.02 ± 134.58	3007.17	73 (8.88)	24.28	0.56 (0.42–0.74) ^****^	0.68 (0.52–0.91) ^**^	0.67 (0.50–0.89) ^**^

PA, physical activity; PA-time, total time of regular participation in any sports or exercise to the point of sweating; HR, hazard ratio; CI, confidence interval; BMI, body mass index; ^*^, *p* < 0.05; ^**^, *p* < 0.01; ^****^, *p* < 0.0001. Model 1 was adjusted for age, sex, drinking, smoking, educational level, marital status, and household income. Model 2 was adjusted for the variables in Model 1 in addition to BMI, hypertension, and diabetes mellitus.

There was no significant relationship between participation in RT and the reduced risk for incident depression in both sexes (Supplementary Table 2). We further investigated the effect of adding RT on depression in participants with low and high leisure-time PA levels (Table 3). For this analysis, the participants were divided into four subgroups based on leisure-time PA levels and RT regularity. RT had no significant effect on depression in either the Low-PA or High-PA groups in both sexes.

**Table 3:** Hazard ratios for new-onset depression according to leisure-time PA levels, regularity of RT, and sex.

	***N***	**Total person-years**	**Participants with depression, *n* (%)**	**Event rate (1,000-person year)**	**PA-time (min/week)**	**RT Levels**			**Crude model,HR (95% CI)**	**Adjusted model,** **HR (95% CI)**
						**Frequency**	**Training period**			
						**(days/week)**	**(month)**	≥**1 year (%)**		
**Total**										
Low-PA	2,048	7207.42	269 (13.13)	37.32	50.39 ± 55.40	-	-	-	1 (reference) ^c^	1 (reference) ^a^
Low-PA+RT	78	261.49	9 (11.54)	34.42	104.11 ± 28.24	3.97 ± 1.66 ^g^	73.87 ± 117.32	66.67 ^f^	0.99 (0.51–1.91)	1.37 (0.70–2.67)
High-PA	1,438	5178.56	129 (8.97)	24.91	299.52 ± 154.97	-	-	-	0.68 (0.55–0.84) ^***^	0.82 (0.66–1.02)
High-PA+RT	403	1466.95	25 (6.20)	17.04	357.53 ± 173.63	4.77 ± 1.44 ^g^	89.68 ± 97.04	84.37 ^f^	0.47 (0.31–0.70) ^***^	0.71 (0.47–1.08)
**Men**										
Low-PA	950	3342.73	102 (10.74)	30.51	43.67 ± 54.59	-	-	-	1 (reference) ^a^	1 (reference)
Low-PA+RT	48	163.85	2 (4.17)	12.21	98.94 ± 31.67	3.90 ± 1.67 ^g^	93.33 ± 139.03	77.08 ^d^	0.41 (0.10–1.67)	0.57 (0.14–2.34)
High-PA	756	2676.42	64 (8.47)	23.91	320.41 ± 179.92	-	-	-	0.79 (0.58–1.09)	0.98 (0.71–1.36)
High-PA+RT	263	961.92	17 (6.46)	17.67	358.05 ± 167.76	4.86 ± 1.52 ^g^	104.87 ± 107.16	87.83 ^d^	0.59 (0.35–0.98) ^*^	0.86 (0.50–1.47)
**Women**										
Low-PA	1,098	3864.68	167 (15.21)	43.21	56.21 ± 55.46	-	-	-	1 (reference) ^c^	1 (reference) ^b^
Low-PA+RT	30	97.65	7 (23.33)	71.69	112.38 ± 19.43	4.10 ± 1.67	42.73 ± 59.94	50.00 ^e^	1.83 (0.86–3.89)	2.02 (0.93–4.40)
High-PA	682	2502.13	65 (9.53)	25.98	276.36 ± 117.34	-	-	-	0.61 (0.46–0.81) ^***^	0.71 (0.52–0.95) ^*^
High-PA+RT	140	505.03	8 (5.71)	15.84	356.57 ± 184.78	4.61 ± 1.27	61.16 ± 65.82	77.86 ^e^	0.37 (0.18–0.75) ^**^	0.53 (0.25–1.10)

PA, physical activity; RT, resistance training; PA-time, total time of regular participation in any sports or exercise to the point of sweating; HR, hazard ratio; CI, confidence interval; BMI, body mass index; ^a^, *p* < 0.05 in the test for trend of HRs; ^b^, *p* < 0.01 in the test for trend of HRs; ^c^, *p* < 0.0001 in the test for trend of HRs; ^d^, *p* < 0.05 compared Low-PA+RT with High-PA+RT; ^e^, *p* < 0.01 compared Low-PA+RT with High-PA+RT; ^f^, *p* < 0.001 compared Low-PA+RT with High-PA+RT; ^g^, *p* < 0.0001 compared Low-PA+RT with High-PA+RT; ^*^, *p* < 0.05; ^**^, *p* < 0.01; ^***^, *p* < 0.001. Adjusted for age, sex, drinking, smoking, educational level, marital status, household income, BMI, hypertension, and diabetes mellitus.

A subgroup analysis was performed for each sex according to age, educational level, household income, BMI, current drinking habit, smoking status, hypertension, and diabetes mellitus. In men, there were no significant associations between high leisure-time PA levels and the reduced risk for incident depression in any subgroup (Supplementary Table 3). In women, the significance of the association between leisure-time PA levels and incident depression was different in some of the subgroups (Supplementary Table 4). Particularly, in the subgroup analyses, the protective benefit of high leisure-time PA levels against depression was significant only in “≥65 years” (*p* < 0.05), “≥high school” (*p* < 0.01), “ < 3 million KRW/month” (*p* < 0.05), “ < 25 kg/m^2^” (*p* < 0.01), “no current drinking” (*p* < 0.05), and “no diabetes mellitus” (*p* < 0.05), respectively.

## Discussion

This study indicated that meeting leisure-time PA recommendations may be associated with protective effects against depression only in women. Interestingly, ≥300 min/week of moderate-intensity leisure-time PA further reduced the risk of incident depression in women. However, RT had no significant effect on depression in either the Low-PA (not meeting the PA guidelines) or High-PA (meeting the PA guidelines) groups in either sex.

Generally, current guidelines recommend all adults to perform moderate-intensity leisure-time PA for at least 150 min per week to reduce depressive symptoms (5, 6). In previous studies, meeting PA recommendations was negatively correlated with the risk of depression in both sexes (7, 22, 23). On the contrary, our finding suggests that satisfying the leisure-time PA guideline is related to reduced depression risk in 33% of women, but not in men, after adjusting for other confounders. This finding is consistent with those of previous studies. Satisfying PA guidelines was negatively associated with the risk of depression in women but not in men (8, 24). Thus, it is unclear whether the protective effects of increased leisure-time PA levels on the risk of depression are the same in both sexes. Several studies have reported that sex-based differences in the antidepressant effects of leisure-time PA depend on training intensity. In women, even relatively low-intensity PA significantly improved mental health by increasing emotional well-being and subjective happiness and decreasing symptoms of somatization, whereas in men, only high-intensity PA markedly improved mental health by enhancing subjective happiness and reducing symptoms of somatization, anxiety, and depression (25, 26). Moreover, in men, substituting the intensity of leisure-time PA from moderate to vigorous-intensity PA for up to approximately 150 min/week was related to a lower likelihood of current depression (27). Taken together, the results indicate that, compared to women, men were likely to prevent depression by performing relatively higher-intensity leisure-time PA, although the abovementioned studies have the limitation of using a cross-sectional design. In our longitudinal study, high levels of moderate-intensity leisure-time PA were not associated with a reduced risk of depression in men. A recent meta-analysis reported the beneficial effect of regular leisure-time PA on the circulating brain-derived neurotrophic factor (BDNF) level, which is decreased in patients with depression, was significant only in women and not in men (28). However, further studies are required as the mechanisms underlying sex-based differences in the antidepressant effects of leisure-time PA have not yet been fully identified. Importantly, the total amount of high-intensity leisure-time PA, which was not considered in our study, should be considered in further longitudinal studies to verify sex-based differences in the antidepressant effects related to regular leisure-time PA.

The potential factors responsible for preventing depression following regular leisure-time PA are improvements in anti-inflammatory activity, neurogenesis, neuroplasticity, self-efficacy, sleep quality, and life satisfaction (29, 30). Some mechanisms that potentially explain the antidepressant effects of regular leisure-time PA have been reported. Specifically, regular PA helps in the prevention and/or management of depressive symptoms by enhancing the BDNF level, which is associated with hippocampal function (31), and increasing the levels of mood-enhancing neurotransmitters such as serotonin and dopamine (32). Particularly, leisure-time PA may be more effective for reducing depressive symptoms than non-leisure-time PA as it enhances pleasant feelings and positive affect and reduces physical exhaustion and negative affect (33). Thus, there is a growing interest in investigating the possibility of a dose–response relationship between the leisure-time PA level and its antidepressant effects. According to a previous study in women, meeting the PA guidelines, but not performing moderate-intensity leisure-time PA for more than 300 min/week, was negatively associated with the risk of depression, whereas performing moderate-intensity leisure-time PA for more than 300 min/week did not show a significant association with depression (9). Furthermore, PA and the risk of depression have a U-shaped dose–response association. Although individuals performing two to three times the recommended PA showed the strongest association with reduced depression, excessive PA increased the risk of depression (22, 34). In women, only a medium level of leisure-time PA was related to a reduced risk of depression, whereas in men, only a low level of leisure-time PA was associated with reduced risk (12). These findings are inconsistent with those of the present study. We investigated whether there was an inverse dose–response association between the amount of leisure-time PA per week and the risk of incident depression. Interestingly, in women, an additional reduction in incident depression associated with ≥300 min/week of moderate-intensity leisure-time PA was observed, after adjusting for other confounders. This finding supports previous studies that reported a dose–response relationship between PA and the risk of depression. There was a graded dose–response pattern between greater improvement in mental health and higher sports activity sessions per week (35) or higher amount of moderate-to-vigorous intensity PA per week (36). Furthermore, cardiorespiratory fitness, an outcome of regular leisure-time PA, showed a clear graded dose–response association with increased emotional well-being and improved depressive symptoms in both sexes (37). Taken together, the inverse dose–response association between leisure-time PA levels and the risk of depression is controversial. Thus, further randomized controlled trials (RCTs) are needed to determine whether there is an optimal intensity, frequency, type, and training period of leisure-time PA to prevent and manage depression.

To the best of our knowledge, despite the current increased interest in muscle-strengthening PA such as RT, the protective effects of RT on depression have received less attention than those of aerobic-related leisure-time PA. In recent cross-sectional studies, performing RT was negatively correlated with the risk of depression in both sexes (16), whereas there was a significant association only in men (38). In a recent RCT of older patients with depression treated with antidepressants, performing 12 weeks of moderate-intensity RT significantly decreased the symptoms of major depressive disorder (39). In another RCT in older individuals with depression, performing 8 weeks of high-intensity RT significantly reduced depressive symptoms compared with standard care, whereas performing 8 weeks of low-intensity RT had no significant beneficial effects (40). Taken together, although the inverse association between regular RT and the risk of depression remains controversial, RT should be performed at moderate or high intensities to manage depression. Furthermore, meta-analytical evidence showed a significant association between performing RT and reduced depressive symptoms regardless of sex (41), but inconsistent results have been reported in more recent studies. Increase in skeletal muscle mass, an outcome of regular RT, showed an inverse association with depressive symptoms in men but not in women (42). In contrast, a 12-week moderate-intensity RT program significantly reduced anxiety and depressive symptoms in older women (43). However, in our observational study, we did not find an association between regular RT and the risk of depression in either sex. This discrepancy may be due to the differences in RT intensity. As the present study evaluated RT regularity using self-reported questionnaires, specific information on RT intensity was unavailable. Therefore, further evaluation is needed to clarify whether there is a significant difference in the antidepressant effect of RT according to the training intensity. Moreover, sex-based differences in the antidepressant effects of RT must be investigated. Thus, further RCTs are required to verify whether there is an optimal intensity, frequency, type, and training period of RT for managing and preventing depression in both sexes.

The major strength of the present study is its longitudinal nature, which investigates PA-related antidepressant effects by simultaneously considering both leisure-time PA levels and participation in RT. Furthermore, we evaluated the cumulative levels of leisure-time PA for 4 years to minimize recall bias, which could occur from a single self-reported questionnaire. However, our study has several limitations. First, as we only included Korean participants, the results may not be applicable to other populations. Second, although the SGDS-K has been validated in the Korean population for screening depression in older adults, the actual incidence of depression may have been underestimated or overestimated. Third, detailed information on occupation-related PA, transportation-related PA, and sedentary behavior were unavailable for the present cohort. Thus, we could not investigate the relationships between them and the risk of incident depression. Lastly, we were unable to obtain detailed information regarding the intensity of RT from self-reported questionnaires; therefore, the association between RT intensity and the risk of incident depression could not be investigated. Therefore, further studies are needed to investigate the optimal frequency, intensity, type, and training period of RT to prevent and manage depression.

In conclusion, our findings suggest that meeting leisure-time PA guidelines may be related to protective effects against depression only in women and not in men. An inverse dose–response association between leisure-time PA levels and the risk of depression was also found only in women, not in men. Furthermore, RT had no significant effect on depression in either sex. However, because training intensity, which plays a crucial role in explaining the antidepressant effects of RT, was not considered in the present study, further RCTs are required to verify the optimal RT intensity for maximizing the beneficial effects on incident depression in both sexes.

## Data availability statement

Publicly available datasets were analyzed in this study. This data can be found at: the Korean Genome and Epidemiology Study (KoGES; 4851-302), Korea National Institute of Health, Korea Disease Control and Prevention Agency.

## Ethics statement

The studies involving human participants were reviewed and approved by the Institutional Review Board Committee of the Korea National Institute of Health, Korea Disease Control and Prevention Agency (Approval No. 2021-04-02-P-A). The patients/participants provided their written informed consent to participate in this study.

## Author contributions

Conceptualization and writing—review and editing: JHP and H-YP. Methodology and resources: H-YP and N-KL. Software and data curation: N-KL. Validation and formal analysis: JHP and N-KL. Investigation, writing—original draft preparation, and visualization: JHP. Supervision, project administration, and funding acquisition: H-YP. All authors read and agreed to the published version of the manuscript.
