# Supplementary figures and images for: Sex-based differences in the association of leisure-time physical activity with the risk of depression: the Ansan and Ansung study of the Korean Genome and Epidemiology Study (KoGES)
Source: Front Public Health. 2023 Jun 15;11:1176879. doi: 10.3389/fpubh.2023.1176879 (PMC10311255; doi:10.3389/fpubh.2023.1176879)

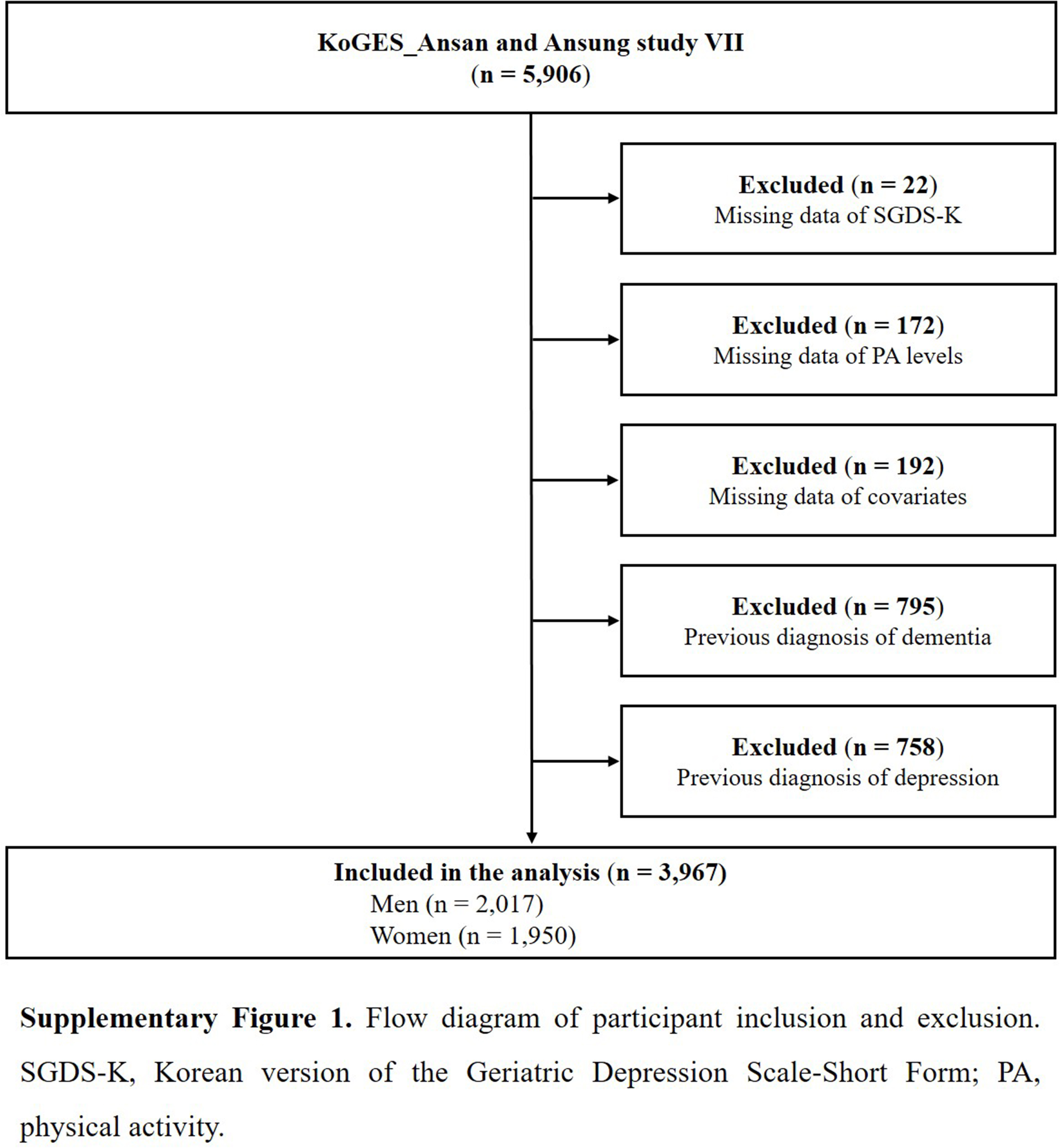

Supplement: Supplementary file 1 [file Image_1.JPEG]
