# Supplementary material for: Sex-based differences in the association of leisure-time physical activity with the risk of depression: the Ansan and Ansung study of the Korean Genome and Epidemiology Study (KoGES)
Source: Front Public Health. 2023 Jun 15;11:1176879. doi: 10.3389/fpubh.2023.1176879 (PMC10311255; doi:10.3389/fpubh.2023.1176879)
Supplement: Supplementary file 2 [file Table_1.DOCX]

**Supplementary Table 1.** Baseline characteristics of participants based on new-onset depression and sex during the follow-up period

| **Variables** | **Men** (n = 2,017) | | ***p*-value** | **Women** (n = 1,950) | | ***p*-value** |
| --- | --- | --- | --- | --- | --- | --- |
|  | **Non-DEP**  (n = 1,832) | **DEP**  (n = 185) |  | **Non-DEP**  (n = 1,703) | **DEP**  (n = 247) |  |
| **Age** (years) | 61.30 ± 7.45 | 63.16 ± 9.01 | < 0.01 | 61.32 ± 7.66 | 64.60 ± 9.12 | < 0.0001 |
| **Marital status**, n (%) |  |  | < 0.01 |  |  | 0.45 |
| Divorced/widowed/single | 80 (4.37) | 16 (8.65) |  | 338 (19.85) | 44 (17.81) |  |
| Married/partnered | 1,752 (95.63) | 169 (91.35) |  | 1,365 (80.15) | 203 (82.19) |  |
| **Educational level**, n (%) |  |  | < 0.0001 |  |  | < 0.0001 |
| ≤ Elementary school | 232 (12.66) | 43 (23.24) |  | 525 (30.83) | 129 (52.23) |  |
| Middle/high school | 1,244 (67.91) | 122 (65.95) |  | 1,073 (63.01) | 113 (45.75) |  |
| ≥ College | 356 (19.43) | 20 (10.81) |  | 105 (6.16) | 5 (2.02) |  |
| **Household income**, n (%) |  |  | < 0.0001 |  |  | < 0.0001 |
| < 1 (million KRW/month) | 296 (16.16) | 57 (30.81) |  | 467 (27.42) | 111 (44.94) |  |
| 1 to <2 | 310 (16.92) | 35 (18.92) |  | 335 (19.67) | 41 (16.60) |  |
| 2 to <3 | 306 (16.70) | 31 (16.76) |  | 286 (16.80) | 36 (14.57) |  |
| 3 to <4 | 303 (16.54) | 17 (9.19) |  | 232 (13.62) | 28 (11.34) |  |
| ≥ 4 | 617 (33.68) | 45 (24.32) |  | 383 (22.49) | 31 (12.55) |  |
| **Drinking habit**, n (%) |  |  | 0.54 |  |  | 0.08 |
| Never drinker | 380 (20.74) | 32 (17.30) |  | 1,274 (74.81) | 201 (81.38) |  |
| Ex-drinker | 231 (12.61) | 25 (13.51) |  | 42 (2.47) | 5 (2.02) |  |
| Current drinker | 1,221 (66.65) | 128 (69.19) |  | 387 (22.72) | 41 (16.60) |  |
| **Smoking habit**, n (%) |  |  | < 0.001 |  |  | 0.45 |
| Never smoker | 457 (24.95) | 28 (15.13) |  | 1,683 (98.83) | 244 (98.79) |  |
| Ex-smoker | 921 (50.27) | 89 (48.11) |  | 6 (0.35) | 2 (0.81) |  |
| Current smoker | 454 (24.78) | 68 (36.76) |  | 14 (0.82) | 1 (0.40) |  |
| **PA-time** (min/week) | 193.05 ± 194.65 | 156.56 ± 182.24 | < 0.05 | 160.96 ± 150.91 | 118.88 ± 140.71 | < 0.0001 |
| **RT**, n (%) | 292 (15.94) | 19 (10.27) | < 0.05 | 155 (9.10) | 15 (6.07) | 0.11 |
| **BMI** (kg/m^2^) | 24.48 ± 2.88 | 23.93 ± 2.84 | < 0.05 | 24.72 ± 3.26 | 24.62 ± 3.40 | 0.64 |
| **WC** (cm) | 87.56 ± 8.34 | 86.81 ± 8.13 | 0.24 | 84.38 ± 9.49 | 86.64 ± 9.74 | < 0.001 |
| **SBP** (mmHg) | 120.47 ± 14.53 | 121.35 ± 14.90 | 0.43 | 118.25 ± 16.50 | 122.96 ± 17.64 | < 0.0001 |
| **DBP** (mmHg) | 78.76 ± 9.58 | 77.74 ± 10.24 | 0.17 | 74.96 ± 9.62 | 76.75 ± 9.40 | < 0.01 |
| **T-Chol** (mg/dL) | 183.12 ± 33.55 | 180.51 ± 32.60 | 0.31 | 194.35 ± 35.08 | 188.98 ± 35.49 | < 0.05 |
| **HDL-C** (mg/dL) | 43.43 ± 11.16 | 43.57 ± 12.07 | 0.87 | 48.20 ± 11.55 | 47.60 ± 10.91 | 0.44 |
| **TG** (mg/dL) | 148.42 ± 109.52 | 144.13 ± 103.48 | 0.61 | 126.35 ± 69.14 | 130.02 ± 82.75 | 0.51 |
| **FBG** (mg/dL) | 99.74 ± 21.41 | 98.99 ± 20.81 | 0.65 | 94.64 ± 19.21 | 95.04 ± 21.33 | 0.78 |
| **Hypertension**, n (%) | 842 (45.96) | 96 (51.89) | 0.12 | 705 (41.40) | 133 (53.85) | < 0.001 |
| **Diabetes mellitus**, n (%) | 338 (18.45) | 30 (16.22) | 0.45 | 263 (15.44) | 42 (17.00) | 0.53 |

DEP, depression; KRW, Korean won; PA-time, total time of regular participation in any sports or exercise to the point of sweating; RT, resistance training; BMI, body mass index; WC, waist circumference; SBP, systolic blood pressure; DBP, diastolic blood pressure; T-Chol, total cholesterol; HDL-C, high-density lipoprotein cholesterol; TG, triglycerides; FBG, fasting blood glucose.
