# Supplementary material for: Sex-based differences in the association of leisure-time physical activity with the risk of depression: the Ansan and Ansung study of the Korean Genome and Epidemiology Study (KoGES)
Source: Front Public Health. 2023 Jun 15;11:1176879. doi: 10.3389/fpubh.2023.1176879 (PMC10311255; doi:10.3389/fpubh.2023.1176879)
Supplement: Supplementary file 3 [file Table_2.DOCX]

**Supplementary Table 2.** Hazard ratios for new-onset depression according to regularity of RT and sex

|  | **N** | **Total person-years** | **Participants with depression**, n (%) | **Event rate** (1,000-person year) | **PA-time** (min/week) | **RT Levels** | | | **Crude Model**,  **HR** (95% CI) | **Adjusted Model**,  **HR** (95% CI) |
| --- | --- | --- | --- | --- | --- | --- | --- | --- | --- | --- |
|  |  |  |  |  |  | **Frequency** | **Training Period** | |  |  |
|  |  |  |  |  |  | (days/week) | (month) | ≥1 year (%) |  |  |
| **Total** |  |  |  |  |  |  |  |  |  |  |
| Non-RT | 3,486 | 12,385.97 | 398 (11.42) | 32.13 | 153.16 ± 163.56 | - | - | - | 1 (reference) | 1 (reference) |
| RT | 481 | 1,728.44 | 34 (7.07) | 19.67 | 316.44 ± 184.72 | 4.64 ± 1.51 | 87.12 ± 100.64 | 81.50 | 0.63 (0.44–0.89) ^**^ | 0.89 (0.62–1.27) |
| **Men** |  |  |  |  |  |  |  |  |  |  |
| Non-RT | 1,706 | 6,019.16 | 166 (9.73) | 27.58 | 166.30 ± 186.82 | - | - | - | 1 (reference) | 1 (reference) |
| RT | 311 | 1,125.76 | 19 (6.11) | 16.88 | 318.06 ± 180.91 | 4.71 ± 1.58 | 103.09 ± 112.49 | 86.17 | 0.62 (0.38–0.99) ^*^ | 0.81 (0.50–1.32) |
| **Women** |  |  |  |  |  |  |  |  |  |  |
| Non-RT | 1,780 | 6,366.82 | 232 (13.03) | 36.44 | 140.56 ± 136.49 | - | - | - | 1 (reference) | 1 (reference) |
| RT | 170 | 602.68 | 15 (8.82) | 24.89 | 313.48 ± 192.00 | 4.52 ± 1.36 | 57.91 ± 65.04 | 72.94 | 0.70 (0.41–1.18) | 1.00 (0.58–1.70) |

RT, resistance training; PA-time, total time of regular participation in any sports or exercise to the point of sweating; HR, hazard ratio; CI, confidence interval; BMI, body mass index; ^*^, *p* < 0.05; ^**^, *p* < 0.01. Adjusted for age, sex, drinking, smoking, educational level, marital status, household income, BMI, hypertension, and diabetes mellitus.
