# Supplementary material for: Sex-based differences in the association of leisure-time physical activity with the risk of depression: the Ansan and Ansung study of the Korean Genome and Epidemiology Study (KoGES)
Source: Front Public Health. 2023 Jun 15;11:1176879. doi: 10.3389/fpubh.2023.1176879 (PMC10311255; doi:10.3389/fpubh.2023.1176879)
Supplement: Supplementary file 4 [file Table_3.DOCX]

**Supplementary Table 3.** Hazard ratios for new-onset depression according to leisure-time PA levels in various subgroups of men

| **Subgroups** | **N** | **Participants with depression**, n (%) | **Leisure-time PA levels** | | | ***p* for interaction** |
| --- | --- | --- | --- | --- | --- | --- |
|  |  |  | **Events** (event rate ^a^) | | **HR** (95% CI)  Low-PA vs. High-PA |  |
|  |  |  | **Low-PA** | **High-PA** |  |  |
| **Age** (years) |  |  |  |  |  |  |
| <65 | 1,373 | 109 (7.94) | 54 (23.10) | 55 (21.12) | 1.06 (0.72–1.58) | 0.31 |
| ≥65 | 644 | 76 (11.80) | 50 (42.78) | 26 (25.15) | 0.86 (0.53–1.40) |  |
| **Educational level** |  |  |  |  |  |  |
| ≤Middle school | 698 | 77 (11.03) | 54 (35.35) | 23 (25.42) | 0.80 (0.48–1.33) | 0.60 |
| ≥High school | 1,319 | 108 (8.19) | 50 (25.26) | 58 (21.22) | 0.99 (0.67–1.46) |  |
| **Household income** |  |  |  |  |  |  |
| <3 (million KRW/month) | 1,035 | 123 (11.88) | 77 (36.60) | 46 (31.33) | 0.93 (0.64–1.36) | 1.00 |
| ≥3 | 982 | 62 (6.31) | 27 (19.25) | 35 (16.13) | 1.00 (0.60–1.67) |  |
| **BMI** (kg/m^2^) |  |  |  |  |  |  |
| <25 | 1,199 | 122 (10.18) | 70 (33.21) | 52 (25.12) | 0.99 (0.68–1.43) | 0.73 |
| ≥25 | 818 | 63 (7.70) | 34 (24.30) | 29 (18.50) | 0.87 (0.52–1.46) |  |
| **Current drinking habit** |  |  |  |  |  |  |
| No | 668 | 57 (8.53) | 32 (27.15) | 25 (21.85) | 1.13 (0.65–1.95) | 0.48 |
| Yes | 1,349 | 128 (9.49) | 72 (30.93) | 56 (22.45) | 0.89 (0.61–1.28) |  |
| **Smoking status** |  |  |  |  |  |  |
| Never | 1,495 | 117 (7.83) | 65 (26.83) | 52 (17.90) | 0.88 (0.60–1.29) | 0.04 |
| Ever | 522 | 68 (13.03) | 39 (35.97) | 29 (39.54) | 1.22 (0.73–2.02) |  |
| **Hypertension** |  |  |  |  |  |  |
| No | 1,079 | 89 (8.25) | 49 (26.46) | 40 (20.15) | 0.94 (0.61–1.46) | 0.77 |
| Yes | 938 | 96 (10.23) | 55 (33.24) | 41 (24.79) | 0.92 (0.60–1.41) |  |
| **Diabetes mellitus** |  |  |  |  |  |  |
| No | 1,649 | 155 (9.40) | 89 (30.14) | 66 (22.60) | 0.93 (0.67–1.30) | 0.76 |
| Yes | 368 | 30 (8.15) | 15 (27.09) | 15 (20.90) | 1.17 (0.53–2.56) |  |

PA, physical activity; HR, hazard ratio; CI, confidence interval; BMI, body mass index; ^a^, event rate presented per 1,000-person years of follow-up. Adjusted for age, sex, drinking, smoking, educational level, marital status, household income, BMI, hypertension, and diabetes mellitus.
