# Supplementary material for: Sex-based differences in the association of leisure-time physical activity with the risk of depression: the Ansan and Ansung study of the Korean Genome and Epidemiology Study (KoGES)
Source: Front Public Health. 2023 Jun 15;11:1176879. doi: 10.3389/fpubh.2023.1176879 (PMC10311255; doi:10.3389/fpubh.2023.1176879)
Supplement: Supplementary file 5 [file Table_4.DOCX]

**Supplementary Table 4.** Hazard ratios for new-onset depression according to leisure-time PA levels in various subgroups of women

| **Subgroups** | **N** | **Participants with depression**, n (%) | **Leisure-time PA levels** | | | ***p* for interaction** |
| --- | --- | --- | --- | --- | --- | --- |
|  |  |  | **Events** (event rate ^a^) | | **HR** (95% CI)  Low-PA vs. High-PA |  |
|  |  |  | **Low-PA** | **High-PA** |  |  |
| **Age** (years) |  |  |  |  |  |  |
| <65 | 1,290 | 123 (9.53) | 73 (29.86) | 50 (21.79) | 0.79 (0.55–1.15) | 0.21 |
| ≥65 | 660 | 124 (18.79) | 101 (66.54) | 23 (32.27) | 0.57 (0.36–0.90) ^*^ |  |
| **Educational level** |  |  |  |  |  |  |
| ≤Middle school | 1,161 | 180 (15.50) | 128 (49.82) | 52 (34.14) | 0.81 (0.58–1.13) | 0.60 |
| ≥High school | 789 | 67 (8.49) | 46 (33.01) | 21 (14.15) | 0.46 (0.27–0.77) ^**^ |  |
| **Household income** |  |  |  |  |  |  |
| <3 (million KRW/month) | 1,276 | 188 (14.73) | 140 (49.06) | 48 (29.38) | 0.70 (0.50–0.97) ^*^ | 1.00 |
| ≥3 | 674 | 59 (8.75) | 34 (30.67) | 25 (18.20) | 0.64 (0.38–1.08) |  |
| **BMI** (kg/m^2^) |  |  |  |  |  |  |
| <25 | 1,120 | 134 (11.96) | 97 (43.67) | 37 (20.63) | 0.58 (0.39–0.86) ^**^ | 0.73 |
| ≥25 | 830 | 113 (13.61) | 77 (44.22) | 36 (29.66) | 0.81 (0.54–1.21) |  |
| **Current drinking habit** |  |  |  |  |  |  |
| No | 1,522 | 206 (13.53) | 148 (46.70) | 58 (26.21) | 0.68 (0.50–0.93) ^*^ | 0.48 |
| Yes | 428 | 41 (9.58) | 26 (32.77) | 15 (18.88) | 0.71 (0.36–1.42) |  |
| **Smoking status** |  |  |  |  |  |  |
| Never | 1,935 | 246 (12.71) | 173 (44.01) | 73 (24.47) | 0.69 (0.52–0.91) ^**^ | - |
| Ever | 15 | 1 (6.67) | 1 (32.15) | 0 (0) | - |  |
| **Hypertension** |  |  |  |  |  |  |
| No | 1,112 | 114 (10.25) | 78 (35.93) | 36 (19.57) | 0.66 (0.44–1.00) | 0.77 |
| Yes | 838 | 133 (15.87) | 96 (53.58) | 37 (31.70) | 0.70 (0.47–1.03) |  |
| **Diabetes mellitus** |  |  |  |  |  |  |
| No | 1,645 | 205 (12.46) | 143 (43.20) | 62 (23.99) | 0.67 (0.49–0.92) ^*^ | 0.76 |
| Yes | 305 | 42 (13.77) | 31 (47.52) | 11 (26.04) | 0.64 (0.32–1.30) |  |

PA, physical activity; HR, hazard ratio; CI, confidence interval; BMI, body mass index; ^a^, event rate presented per 1,000-person years of follow-up; ^*^, *p* < 0.05; ^**^, *p* < 0.01. Adjusted for age, sex, drinking, smoking, educational level, marital status, household income, BMI, hypertension, and diabetes mellitus.
